# The Relationship Between Frailty and Clinical and Patient-Reported Outcomes After Hip or Knee Arthroplasty: A Retrospective Cohort Study

**DOI:** 10.5435/JAAOSGlobal-D-22-00249

**Published:** 2023-01-12

**Authors:** Christine M. McDonough, Stefanie C. Altieri Dunn, Andrew Bilderback, Adolph J. Yates, MaCalus V. Hogan, Daniel E. Hall

**Affiliations:** From the Physical Therapy School of Health and Rehabilitation Sciences University of Pittsburgh (McDonough)Quality Data Analytics Expert (Dunn), The Wolff Center at UPMC, Pittsburgh, PA; the Manager (Bilderback), Quality Analytics, The Wolff Center at UPMC, Pittsburgh, PA; the Department of Orthopaedic Surgery (YatesJr.), University of Pittsburgh School of Medicine; the UPMC-Shadyside Hospital (Yates), Shadyside Medical Building, Pittsburgh, PA; the Orthopaedic and Musculoskeletal Services UPMC Health Plan (Hogan), Department of Orthopaedic Surgery, University of Pittsburgh School of Medicine – UPMC; and the Department of Surgery (Hall), University of Pittsburgh, The Wolff Center at UPMC, Pittsburgh, PA.

## Abstract

Frailty is associated with increased risks related to surgery. There is emerging consensus that assessment of these risks should include frailty, yet little is known regarding the relationship between prospective frailty measurement and clinical and patient-reported outcomes.

**Methods:**

This retrospective cohort study included adults having hip or knee arthroplasty in one health system between April 2016 and April 2021 within a quality improvement initiative to identify frail adults and support preoperative optimization of care and outcomes. The Risk Analysis Index (RAI) was completed, and scores were available at the time of initial consultation. Scores ≤ 29 were considered robust, 30 to 36 normal, 37 to 44 frail, and ≥ 45 very frail. The Hip Disability and Osteoarthritis Outcome Score-Joint Replacement (HOOS-JR) or Knee Injury and Osteoarthritis Outcome Score-Joint Replacement (KOOS-JR) was administered for the affected joint at preoperative and postoperative clinic visits as well as the patient-acceptable symptom state (PASS) and global rating of change. Patients were included if they had diagnosis-related group (DRG) codes for primary (DRG 469, 470) or bilateral (DRG 461, 462) joint arthroplasty, a completed postoperative HOOS-JR or KOOS-JR, and a preoperative RAI score recorded no more than 270 days before the eligible arthroplasty procedure. Postoperative periods were defined as 0 to 3 months and > 3 months.

**Results:**

Among 3350 individuals, the mean age for those with hip and knee arthroplasty was 64 and 67 years, respectively. RAI score–based frailty level was not associated with postoperative HOOS-JR and KOOS-JR score change at 0 to 3 months or > 3 months, % reaching substantial clinical benefit, global rating of change, or PASS at either time point. Frailty as measured by RAI was associated with longer hospital length of stay and 30-day but not 7-day readmission.

**Conclusion:**

These results suggest that frail patients can and do achieve similar outcomes compared with their more robust counterparts.

Over a million total hip and knee arthroplasties are done annually in the United States,^1^ with rates increasing because of an aging population and wider access. The increasing majority of these procedures are for patients older than 65 years, raising important questions about outcomes within a geriatric population with an increased incidence of comorbid conditions and decreasing functional mobility. Given that joint arthroplasty for osteoarthritis is considered elective, incorporating probabilities of outcomes tailored to a patient's risk profile enhances the shared decision-making process.

There is evidence that a substantial proportion of individuals do not achieve full functional recovery at or beyond one year after arthroplasty.^2-4^ In particular, older individuals with characteristics of frailty are at higher risk of poor outcomes after any surgery including hip or knee arthroplasty.^5-7^ Although risks of death and poor postoperative outcomes increase with age, there is evidence that other factors are important predictors of postsurgical outcomes, including, physical function, comorbid conditions, and frailty.

Frailty is a concept used in geriatrics and beyond to identify individuals with decreased physiological reserve that puts them at risk of death.^8,9,10,11^ Frailty is associated with substantially increased risk of complications, readmissions, death, and poor functional recovery from surgery.^6,12-14^ Therefore, there is emerging consensus that an assessment of frailty should be among the risks that inform decision making for elective surgery. Although controversy persists regarding how frailty is conceptualized, defined, and measured,^15^ a variety of validated frailty metrics exist that are suitable for resource-intensive research protocols or retrospective adjustment of registry and administrative data.^12,16,17,18,19^ The Risk Analysis Index (RAI) is particularly suitable for at-scale frailty screening across predominantly robust populations.^20^ The RAI uses a weighted scale to render a score (zero to 82) indicating increased risk of postoperative mortality based on age, sex, living location, appetite, weight loss, cognitive decline, activities of daily living, dyspnea, congestive heart failure, renal insufficiency, and malignant cancer. It can be calculated by clinicians or through patient self-report, and it has been widely validated in multiple populations to establish thresholds representative of notable postoperative risk. Although the RAI is associated with orthopaedic outcomes, generally,^6^ the literature is sparse regarding RAI and joint arthroplasty surgery, particularly its association with patient-reported outcomes (PROs).^21^ Much of the published research to date on frailty and joint arthroplasty, based on retrospective ascertainment from administrative data, has been focused on clinical and operational outcomes such as death, complications, readmissions, and length of stay (LOS).^21^ Little is known about joint arthroplasty outcomes when frailty is used prospectively to inform clinical decision making and its effect on patient functioning.

Capturing improvement in PROs after lower extremity arthroplasty has become a new priority for payers. Medicare has developed and plans to institute both hospital and surgeon-specific performance measures that capture percentile rankings of the percentages of patients achieving substantial clinical benefit based on Hip Disability and Osteoarthritis Outcome Score-Joint Replacement (HOOS-JR) and Knee Injury and Osteoarthritis Outcome Score-Joint Replacement (KOOS-JR) scores.^22,23^

Therefore, in this study, we aimed to explore the relationship between preoperative RAI score and postoperative functioning, perceived improvement, satisfaction with symptoms, complications, and care process measures including hospital LOS and readmission.

## Methods

### Study Design and Ethical Oversight

This is a retrospective, longitudinal cohort study of adults having hip or knee arthroplasty between April 2016 and April 2021 in the context of an ongoing quality improvement initiative in one multihospital health system. All work was approved by the institutional Quality Review Committee as a quality improvement project. Data are reported according to STROBE standards for observational study reporting excellence.^24^

### Selection of Frailty Instrument

Frailty indices can be calculated retrospectively using surgical registries or administrative data. Our health system leadership considered an automated frailty index using preexisting administrative data, but the lead time required to develop and implement it was considered too long. Furthermore, such a solution would not render a score for patients with no previous data in the electronic health record (EHR), and thus, it was determined that a point-of-care instrument was required. Health system leadership selected the RAI because, at the time, it was the only tool proven feasible for routine, real-time, point-of-care assessment in predominantly robust populations.^25,26^ Based on data from our health system and elsewhere, the RAI has subsequently emerged among the most thoroughly validated measures of surgical frailty,^5-7^ and the only one with demonstrated feasibility for system-wide implementation at the point of care,^20,27^ taking a median of 30 seconds to complete without disrupting clinical flow. Therefore, the RAI was selected as the measure of surgical frailty and captured for all surgical specialties across our health system. It was implemented at the beginning of July 2016 and subsequently automated into the EHR, with more than 400,000 assessments to date.

### Sample and Setting

At the beginning of July 2016, eight surgical specialties within the five-hospital system began assessing frailty with the RAI for all new and preoperative patients, recording the RAI value in the EHR. Details of this project were published in 2020.^20,27,28^ Briefly, the surgical specialties, including orthopaedic surgery comprised academic and nonacademic physician practices in urban, suburban, and rural settings. The project aimed to identify frail individuals and support presurgical decision making to optimize care and outcomes. A best-practice alert was provided in the EHR that prompted providers to indicate whether surgery was under consideration, and if so, for patients with elevated RAI scores, providers were prompted to (1) attest to documentation of a shared decision-making process informed by frailty-associated risks or (2) arrange referral for additional frailty assessment and management to the patient's primary care provider or an existing interdisciplinary preoperative clinic aimed at mitigating manipulable risks. The program was successful in capturing RAI on ≥ 80% of all eligible patients by December 2016. As part of usual care, patient-reported measures were administered. Either HOOS-JR or KOOS-JR was administered for the affected joint at preoperative and postoperative clinic visits for patients scheduled for hip or knee arthroplasty. Although the system hospitals in this study were required to participate in the Comprehensive Care for Joint Replacement Model (CJR), patients from all payers were included in data acquisition, not just fee-for-service Medicare. In addition, at postoperative visits, the global rating of change (GRC) and patient acceptable symptom state (PASS) were administered. The billing data and the inpatient electronic medical record were used to extract patient demographics, comorbidities, procedure details, readmissions, and HOOS-JR and KOOS-JR scores. Patients were identified using diagnosis-related group (DRG) codes for primary arthroplasty (DRG 469, 470) and bilateral (DRG 461, 462) joint arthroplasty. We used existing data indicating patient priority level to identify whether surgery was elective, urgent, or originating from the emergency department or trauma center.

Inclusion criteria were that patient records must contain the following: (1) a completed HOOS-JR or KOOS-JR PRO survey and (2) a preoperative RAI score recorded no more than 270 days before the eligible arthroplasty procedure. The preoperative PRO was included if it was completed within 90 days before the procedure and any postoperative HOOS-JR or KOOS-JR PRO was included. GRC and PASS were included if they were completed on the same date as the HOOS-JR or KOOS-JR. Patient records were excluded if the PRO laterality did not match that of the recorded joint arthroplasty.

#### Follow-up Periods

Postoperative periods were defined as zero to 3 months and greater than 3 months. For patients with >1 PRO in the defined interval, only the PRO with the highest score was used.

### Measures and Outcomes

#### KOOS-JR

The KOOS-JR^29-31^ is a self-reported measure of symptoms and function related to total knee arthroplasty for osteoarthritis derived from the parent measure, the KOOS.^32^ It consists of seven questions framed within the past week: one on stiffness, four on pain with activities, and two on difficulty with activities. Responses are coded from none (zero) to extreme (4) stiffness, pain or difficulty, and values are summed to a score from zero to 28. Scores were reversed and transformed so that zero represents total disability and 100 represents perfect knee health. Distribution-based minimum detectable change estimates with 80% to 95% confidence limits for the KOOS-JR were 7 to 11 points, and minimum clinically important difference (MCID) estimates ranged from 6 to 20.^31^ Point estimates for MCID and substantial clinical benefit (SCB) were 14 and 20 points, respectively.^31^

#### Hip Disability and Osteoarthritis Outcome Score-Joint Replacement

HOOS-JR^33^ is a self-reported measure of symptoms and function related to total hip arthroplasty for osteoarthritis derived from the parent measure, the HOOS.^34^ It consists of six questions framed within the past week: two on pain with activities and four on difficulty with activities. Responses are coded from none (zero) to extreme (4), pain or difficulty, and values are summed to a score from zero to 24. Scores are reversed and transformed so that zero represents total disability and 100 represents perfect hip health. Distribution-based minimum detectable change estimates with 80% to 95% confidence limits were 8 to 11 points, and MCID estimates ranged from 7 to 22.^31^ Anchor-based MCID and SCB estimates were 18 and 22 points, respectively.^31^

### The Risk Analysis Index

As described above, the RAI is calculated based on a 14-item survey. In this cohort, the survey was given to patients as they checked in to the surgeon's clinic. A medical assistant then transferred patient responses to the electronic record to compute and record the RAI score along with other vital signs. This approach took a median 30 seconds to complete, did not disrupt clinical workflow, and provided the surgeon with a score at the time of initial consultation that subsequently informed decision making. The medical assistant or the surgeon was free to adjust patient responses to better reflect findings from their clinical history and examination. Scores < 30 are considered robust, 30 to 36 normal, 37 to 44 frail, and ≥ 45 very frail.

### Patient Acceptable Symptom Score

The PASS is meant to measure the threshold of acceptable treatment outcome from the patient's perspective. It comprises a single question. In this study, the question was, “Taking into account all the activities you have during your daily life, your level of pain, and also your functional impairment, do you consider the current state of your hip/knee to be satisfactory?” with potential responses being “yes” or “no.”^35^

### Global Rating of Change

The GRC addresses the degree of improvement related to treatment from the patient's perspective.^36^ Often framed as a single question, in this study, it was, “Overall, how would you rate the change in the status of your hip/knee since you first saw this physician for your current problem?” The response options included very much worse, much worse, somewhat worse, a little worse, no change, a little better, somewhat better, much better, and very much better.

### Hospital Length of Stay and Readmissions

The outcome of hospital LOS was determined based on admission and discharge dates of the index procedure. Readmission hospitalizations were calculated at intervals of 7, 30, and 90 days from the date of discharge of the index hospitalization.

### Statistical Analysis

Continuous variables were summarized as mean and standard deviation and were analyzed using the Kruskal-Wallis equality-of-populations rank test. Categorical variables were summarized using percentages and analyzed using the likelihood ratio chi square test. Scores for measure were calculated as described above, and mean score change between time points was calculated. The relationship between frailty and patient-reported outcomes was further assesses by calculating the proportion of patients who achieved clinically meaningful change using three approaches. MCID and SCB were calculated based on published anchor-based estimates as described above.^31^ MCID was also calculated based on the score distribution of the sample using 0.5 standard deviation of the preoperative scores.^37^ All statistical analyses were conducted using Stata 16.0 software (StataCorp LLC, College Station, Texas).

## Results

**Figure 1:**
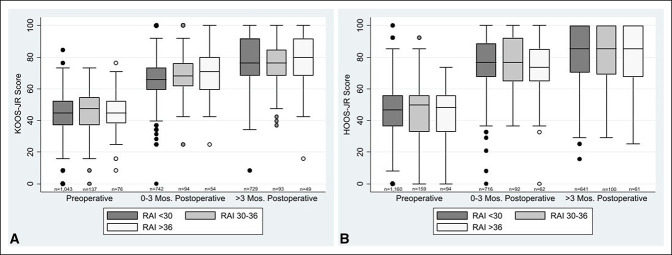
**A,** Graph showing preoperative and postoperative KOOS-JR scores. **B,** Graph showing preoperative and postoperative HOOS-JR scores. HOOS-JR = Hip Disability and Osteoarthritis Outcome Score-Joint Replacement; KOOS-JR = Knee Injury and Osteoarthritis Outcome Score-Joint Replacement

**Figure 2:**
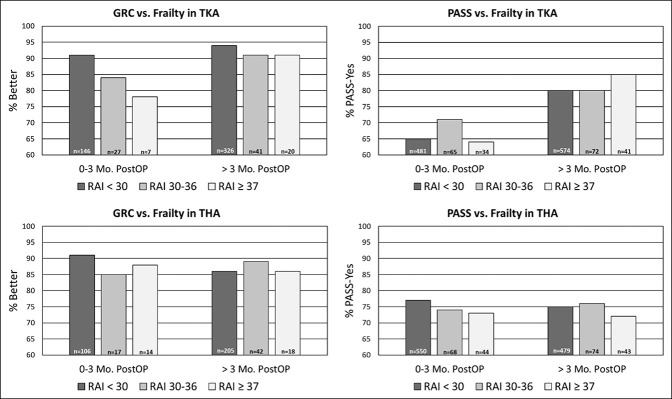
Graph showing postoperative global rating of change (GRC) and patient acceptable symptom state (PASS) by RAI frailty category for total knee arthroplasty(TKA) and total hip arthroplasty (THA). For the GRC, % better is defined by the proportion of patients with responses of very much better, much better, and somewhat better. RAI = Risk Analysis Index

Our cohort included 1592 individuals who underwent knee surgery and 1758 who underwent hip surgery between April 2016 and April 2021 and for whom RAI was collected. Average days for the follow-up measurement of HOOS-JR and KOOS-JR were 315 and 343 days, respectively. The median age for those with knee arthroplasty was 67 years (range: 35.4 to 95.4), and 57% were women and 89% were White (Tables 1 and 2). Additional details are presented in Supplemental Tables 1 and 2, http://links.lww.com/JG9/A254. Patients who underwent hip arthroplasty had a median age of 64.6 years (range: 20 to 97.5), and 52% were female. Of the 1758 *total hip arthroplasty* procedures, 1723 (98.0%) were elective, 21 (1.9%) were from the Emergency Department, 7 (0.4%) were from the trauma center, and 7 (0.4%) were urgent. Consistent with other surgical populations, 6% of individuals with knee arthroplasty and 7% with hip arthroplasty had RAI scores ≥37 indicative of frailty, and an increased RAI score was associated with increased age and sex. For both hip and knee arthroplasties, more than 60% of the frail patients were men. We were not able to assess the effect of race because of limited numbers, especially in the higher frailty categories. RAI score was not associated with postoperative function as measured by the HOOS-JR and KOOS-JR at zero to 3 months or at greater than 3-month follow-up (Tables 3 and 4 and Figure 1). RAI score was also not associated with higher or lower percentage rates of achieving substantial clinical benefit (Tables 5 and 6). Although there was a trend toward fewer individuals above the risk threshold reporting improvement on the GRC zero to 3 months after knee arthroplasty, and a similar pattern for hip arthroplasty, this did not reach statistical significance and was not evident at the follow-up after 3 months (Supplemental Tables 3, http://links.lww.com/JG9/A250, and 4, http://links.lww.com/JG9/251). No association was observed between RAI score and PASS at either time point (Supplemental Tables 5, http://links.lww.com/JG9/A252, and 6, http://links.lww.com/JG9/A253). No differences were observed in the proportion of patients who reported improvement (including responses of very much better, much better, and somewhat better) on the GRC (Figure 2) or who achieved MCID or SCB by frailty level (Tables 5 and 6).

**Table 1:** Characteristics of the Total Knee Arthroplasty Sample

Factor or Variable	Total Knee Arthroplasty			
	Overall	RAI < 30	RAI 30–36	RAI ≥ 37
n (%)	1592	1323 (83.1)	172 (10.8)	97 (6.1)
Age, mean (SD)	67.1 (8.9)	66.0 (8.7)	71.1 (9.2)	73.8 (6.9)
Female, n (%)	909 (57.1)	769 (58.1)	106 (61.6)	34 (35.1)
Race, n (%)				
White	1416 (88.9)	1179 (89.1)	151 (87.8)	86 (88.7)
Black	145 (9.1)	118 (8.9)	18 (10.5)	9 (9.3)
Other	31 (2.0)	26 (2.0)	3 (1.7)	2 (2.1)
Died	0 (0)	0 (0)	0 (0)	0 (0)

RAI = Risk Assessment Index

**Table 2:** Characteristics of the Total Hip Arthroplasty Sample

Factor or Variable	Total Hip Arthroplasty			
	Overall	RAI < 30	RAI 30–36	RAI ≥ 37
n (%)	1758	1423 (80.9)	207 (11.8)	128 (7.3)
Age, mean (SD)	64.1 (11.7)	62.3 (11.1)	71.4 (10.9)	73.1 (10.3)
Female, n (%)	922 (52.5)	760 (53.4)	114 (55.1)	48 (37.5)
Race, n (%)				
White	1551 (88.2)	1246 (87.6)	187 (90.3)	118 (92.2)
Black	179 (10.2)	150 (10.5)	20 (9.7)	9 (7.0)
Other	28 (1.6)	27 (1.9)	0 (0)	1 (0.8)
Died	3 (0.2)	1 (0.1)	1 (0.5)	1 (0.8)

RAI = Risk Assessment Index

**Table 3:** Comparison of KOOS-JR Scores and RAI Frailty Status^a^

Factor or Variable	KOOS-JR Scores					
	RAI<30		RAI 30–36		RAI ≥ 37	
	n	Mean (SD)	n	Mean (SD)	n	Mean (SD)
Preoperative	1043	44.7 (13.5)	137	45.2 (13.4)	76	45.8 (12.5)
Postoperative						
0–3 months	742	67.6 (13.8)	94	69.4 (13.6)	54	69.1 (15.4)
> 3 months	729	78.7 (16.0)	93	76.6 (16.3)	49	79.0 (19.4)
Change score						
0–3 months	565	22.3 (17.8)	67	23.8 (16.8)	43	24.2 (18.5)
>3 months	546	33.9 (19.6)	67	32.2 (17.4)	37	34.8 (20.9)

KOOS-JR = Knee Injury and Osteoarthritis Outcome Score-Joint Replacement; RAI = Risk Analysis Index

a No comparisons between mean KOOS-JR scores for RAI frailty status were statistically significant at the *P* < 0.05 level.

**Table 4:** Comparison of HOOS-JR Scores and RAI Frailty Status^a^

Factor or Variable	HOOS-JR Scores					
	RAI<30		RAI 30–36		RAI ≥ 37	
	n	Mean (SD)	n	Mean (SD)	n	Mean (SD)
Preoperative	1160	45.0 (15.5)	159	44.3 (19.6)	94	43.1 (17.4)
Postoperative						
0–3 months	716	76.1 (15.9)	92	76.9 (16.0)	62	73.6 (19.3)
> 3 months	641	82.1 (17.8)	100	81.9 (18.9)	61	80.6 (19.0)
Change score						
0–3 months	552	30.4 (19.5)	63	32.1 (21.8)	41	31.2 (24.9)
>3 months	480	37.9 (20.7)	71	39.6 (26.3)	40	38.4 (23.6)

HOOS-JR = Hip Disability and Osteoarthritis Outcome Score-Joint Replacement; RAI = Risk Analysis Index

a No comparisons between mean HOOS-JR scores for RAI frailty status were statistically significant at the *P* < 0.05 level.

**Table 5:** Relationship Between RAI Score–based Frailty Level and Proportion of Total Knee Arthroplasty Patients Achieving Minimal and Substantial Clinical Benefit

Clinical Benefit Approach	Postsurgery Time Frame (months)	Total Knee Arthroplasty			
		RAI < 30	RAI 30–36	RAI ≥ 37	*P*
		Clinical Benefit N (%)	Clinical Benefit N (%)	Clinical Benefit N (%)	
Percentage of patients who achieved substantial clinical benefit (SCB)^a^	0–3	291 (51.5)	38 (56.7)	28 (65.1)	0.177
	>3	418 (76.6)	50 (74.6)	29 (78.4)	0.904
Percentage of patients who achieved minimal clinically important difference (MCID)^b^	0–3	368 (65.1)	46 (68.7)	31 (72.1)	0.567
	>3	467 (85.5)	57 (85.1)	30 (81.1)	0.774
Percentage of patients who achieved MCID based on ≥ 0.5 standard deviation of preoperative scores^c^	0–3	465 (82.3)	57 (85.1)	34 (79.1)	0.718
	>3	500 (91.6)	62 (92.5)	33 (89.2)	0.846

HOOS-JR = Hip Disability and Osteoarthritis Outcome Score-Joint Replacement; KOOS-JR = Knee Injury and Osteoarthritis Outcome Score-Joint Replacement; RAI = Risk Analysis Index

a SCB = KOOS-JR change score ≥20 or HOOS-JR change score ≥22.

b MCID = KOOS-JR change score ≥14 or HOOS-JR change score ≥18.

c 0.5 standard deviation of preoperative mean for KOOS-JR for RAI groupings RAI < 30, RAI 30 to 36, and RAI ≥37 was 6.75, 6.70, and 6.25, respectively. 0.5 SD of preoperative mean for HOOS-JR for RAI groupings RAI < 30, RAI 30 to 36, and RAI ≥37 was 7.75, 9.80, and 8.70, respectively.

**Table 6:** Relationship Between RAI Score–based Frailty Level and Proportion of Total Hip Arthroplasty Patients Achieving Minimal and Substantial Clinical Benefit

Clinical Benefit Approach	Postsurgery Time Frame (months)	Total Hip Arthroplasty			
		RAI < 30	RAI 30–36	RAI ≥ 37	*P*
		Clinical Benefit, N (%)	Clinical Benefit, N (%)	Clinical Benefit, N (%)	
Percentage of patients who achieved substantial clinical benefit (SCB)^a^	0–3	357 (64.7)	42 (66.7)	22 (53.7)	0.343
	>3	373 (77.7)	53 (74.7)	28 (70.0)	0.501
Percentage of patients who achieved minimal clinically important difference (MCID)^b^	0–3	391 (70.8)	47 (74.6)	26 (63.4)	0.475
	>3	402 (83.8)	56 (78.9)	32 (80.0)	0.537
Percentage of patients who achieved MCID based on ≥ 0.5 standard deviation of preoperative scores^c^	0–3	498 (90.2)	51 (81.0)	38 (92.7)	0.088
	>3	442 (92.1)	60 (84.5)	34 (85.0)	0.074

HOOS-JR = Hip Disability and Osteoarthritis Outcome Score-Joint Replacement; KOOS-JR = Knee Injury and Osteoarthritis Outcome Score-Joint Replacement; RAI = Risk Analysis Index

a SCB = KOOS-JR change score ≥20 or HOOS-JR change score ≥22.

b MCID = KOOS-JR change score ≥14 or HOOS-JR change score ≥18.

c 0.5 standard deviation of preoperative mean for KOOS-JR for RAI groupings RAI < 30, RAI 30 to 36, and RAI ≥37 was 6.75, 6.70, and 6.25, respectively. 0.5 SD of preoperative mean for HOOS-JR for RAI groupings RAI < 30, RAI 30 to 36, and RAI ≥37 was 7.75, 9.80, and 8.70, respectively.

For individuals with knee arthroplasty, hospital LOS and 90-day readmission were associated with RAI score, whereas 7-day and 30-day readmissions were not (Tables 7 and 8). For those with hip arthroplasty, RAI score was associated with hospital LOS and 30-daybut not 7-day readmission. The relationship between RAI score and 90-day readmission approached but did not reach statistical significance for this group.

**Table 7:** Operational Outcomes for the Total Knee Arthroplasty Sample (n = 1592)

Factor or Variable	Total Knee Arthroplasty				
	Overall	RAI < 30	RAI 30–36	RAI ≥ 37	*P*
Hospital LOS, days, mean (SD)	2.3 (1.3)	2.2 (1.3)	2.7 (1.6)	2.5 (1.4)	0.001
Readmissions					
7-day	26 (1.6)	20 (1.5)	2 (1.2)	4 (4.1)	0.220
30-day	56 (3.5)	41 (3.1)	7 (4.1)	8 (8.3)	0.063
90-day	95 (6.0)	68 (5.1)	17 (9.9)	10 (10.3)	0.015

LOS = length of stay; RAI = Risk Analysis Index

**Table 8:** Operational Outcomes for the Total Hip Arthroplasty Sample (n = 1758)

Factor or Variable	Total Hip Arthroplasty				
	Overall	RAI < 30	RAI 30–36	RAI ≥ 37	*P*
Hospital LOS, days, mean (SD)	2.1 (1.8)	2.0 (1.6)	2.6 (2.2)	3.2 (2.4)	<0.001
Readmissions					
7-day	22 (1.3)	16 (1.1)	5 (2.4)	1 (0.8)	0.326
30-day	63 (3.6)	42 (3.0)	11 (5.3)	10 (7.8)	0.016
90-day	123 (7.0)	91 (6.4)	16 (7.7)	16 (12.5)	0.053

LOS = length of stay; RAI = Risk Analysis Index

## Discussion

This study of 3350 individuals who underwent hip or knee surgery in one health system between April 2016 and April 2021 found that RAI score was associated with hospital LOS and readmission but not functional outcomes. These data suggest that frail patients can and do achieve similar outcomes compared with their more robust counterparts. Although our analysis did not evaluate the differences in interventions for these two groups, we think that this may be due to adjunctive management strategies aimed at mitigating the frailty-associated risks identified by the RAI or possibly a decision to forego surgery. For example, in our quality improvement initiative, elevated RAI scores mandate either or both (1) referral to a multidisciplinary preoperative clinic aimed at mitigating manipulable risks or (2) a robust shared decision-making process informed by frailty-associated risks to ensure goal-concordant treatment plans.

The longer LOS and more frequent readmission found in the current study is as would be expected in this higher risk cohort that is vulnerable to postoperative complications. This is consistent with other data of similarly frail patients, but whereas such complications and readmissions can sometimes result in patient death (e.g., “failure to rescue”) in this cohort, the care rendered in these longer and more frequent admissions achieved the goal of outcomes equivalent to more robust counterparts. Our finding of longer LOS for those who were frail should inform perioperative planning for providers, patients, and families.

Our results are consistent with existing literature using retrospective analyses that have found a relationship between frailty and medical, operational, and cost outcomes such as increased readmissions and hospital LOS.^38^ However, our approach differs from most of the published literature in that measurement of frailty was conducted prospectively and made available to inform clinical decision making to optimize clinical outcomes. Our results are consistent with those of similar studies,^26,38-40^ indicating that measurement of frailty used to inform clinical care is associated with similar medical and operational outcomes. Our study adds to the literature addressing functional outcomes and is consistent with Meessen et al.,^41^ who found that although frail patients had lower functioning than their more robust counterparts, their change in functioning after arthroplasty was similar in magnitude.

Although this quality improvement study was not designed to generate generalizable knowledge, the system-wide implementation across multiple hospitals and a wide range of age and conditions suggests that similar results could be obtained in similar practice environments. We did not exclude patients younger than 65 years or those with bilateral arthroplasty or arthroplasty for specific diseases or conditions, such as fragility fractures, cancer, or trauma. This is consistent with the aims of the project to address frailty for all individuals undergoing surgery. Nevertheless, the large majority of the sample represented elective arthroplasty.

### Limitations

It is unclear whether the relatively favorable outcomes for frail individuals demonstrated here would be observed in all surgical practices and are constitutive of the disease process and method of treatment—or whether these favorable findings are confounded by the concurrent quality improvement initiative. Preliminary data demonstrate that across all procedure types, a general improvement was observed in postoperative mortality after implementing the RAI-triggered “surgical pause,”^27^ but data specific to orthopaedic or joint arthroplasty are not available. In addition, our data do not account for potential differences in postacute care, which may have affected our longer-term outcomes.

Ideally, measurement of knee and hip-specific functioning would be conducted at multiple specific time points. However, because these measures were administered within the context of clinical care, there was variation in their timing. Although this limits our ability to fully understand the trajectory of recovery with usual care, it is sufficient to address our main research question. Although the HOOS-JR and KOOS-JR focus on hip and knee-specific functioning, the limited number of items included in short forms may have resulted in limited sensitivity.

Although our sample was drawn from multiple hospitals with a wide range of age, frailty, condition, and procedure, it did not comprise a nationally representative sample of either adults undergoing arthroplasty or of provider settings. Therefore, caution is warranted in generalizing these findings to other settings, perhaps especially in settings with more racial and ethnic diversity. For example, a recent retrospective analysis of a national sample found that race and ethnicity moderated the effect of frailty on outcomes of hip and knee arthroplasty and that this relationship was strongest for White non-Hispanic adults.^42^

### Future Research

Future research should be conducted with a larger sample and specific measurement time points covering the continuum of recovery to clarify functional trajectories across the range of RAI scores. These and similar published results support a prospective trial using more sensitive measures of hip- and knee-specific outcomes, measuring interventions and other downstream actions from the frailty assessment. Finally, longer-term studies should be conducted to investigate whether joint arthroplasty could have a positive effect on frailty status through a mechanism of improved mobility—a variable often included in frailty assessments.

## Conclusions

The RAI seems to be useful in identifying individuals at risk for increased LOS and readmissions. Notwithstanding potential selection bias, this study provides evidence that joint arthroplasty can be safely applied in this population and that frailty is not an absolute contraindication.
